# Imaging of Fiber Waviness in Thick Composites with Unknown Material Properties Using Probability-Based Ultrasound Non-Reciprocity

**DOI:** 10.3390/ma16103786

**Published:** 2023-05-17

**Authors:** Zhen Zhang, Andong Cao, Qian Li, Weidong Yang, Yan Li

**Affiliations:** School of Aerospace Engineering and Applied Mechanics, Tongji University, Shanghai 200092, China; 2013_tjzhangzhen@tongji.edu.cn (Z.Z.); 2210151@tongji.edu.cn (A.C.); 20501@tongji.edu.cn (W.Y.)

**Keywords:** ultrasonic testing, fiber waviness, porosity, phased array, defect characterization

## Abstract

Fiber waviness and voids may be produced in thick composites due to improper manufacturing conditions and consequently pose a risk of structural failure. A proof-of-concept solution for imaging fiber waviness in thick porous composites was proposed from both numerical and experimental studies, via calculating ultrasound non-reciprocity along different wave paths in a sensing network constructed by two phased array probes. Time-frequency analyses were conducted to reveal the cause of ultrasound non-reciprocity in wavy composites. Subsequently, the number of elements in the probes and excitation voltages was determined for fiber waviness imaging using the ultrasound non-reciprocity with a probability-based diagnostic algorithm. The fiber angle gradient was observed to cause ultrasound non-reciprocity and fiber waviness in the thick wavy composites were successfully imaged regardless of presence of voids. This study proposes a new feature for the ultrasonic imaging of fiber waviness and is expected to contribute to processing improvement in thick composites without prior knowledge of material anisotropy.

## 1. Introduction

Thick carbon-fiber-reinforced plastic (CFRP) components have been increasingly adopted in aerospace structures due to their excellent static and fatigue properties [1]. The key factors limiting the application range of large thick CFRP are design–manufacturing compliance and manufacturing reliability [2]. Mixed-type defects including fiber waviness and voids may be produced in the thick CFRP due to uneven pressure and temperature, which introduce the risk of structural failure [3].

Non-destructive testing (NDT) is prevailingly used for the quality verification and damage evaluation of composite structures after manufacturing and during service [4,5]. X-ray computing tomography and ultrasound testing (UT) [6,7,8] are the two main NDT technologies for the quality verification of thick CFRP, and the latter is more suitable for the in-situ inspection of large components [7,9]. UT, using the changes in reflected and transmitted ultrasound, was already well developed to detect volumetric defects (e.g., voids, delamination and cracks) in composite structures [10,11,12,13,14,15]. Zhu [16] adopted a loading-assisted diffuse wave inspection of delamination in a unidirectional composite. The delamination geometry was imaged through Bayesian inference together with numerical decorrelation coefficients. Li [17] proposed a self-interference cancellation algorithm for defect location in composites with minimized interfacial reflections based on a matching pursuit algorithm. Liu [18] proposed a time-corrected ultrasonic total focusing method to comprehensively consider the influence of structural anisotropy, inhomogeneity and probing frequency on wave velocity, and successfully imaged defects in thick composites over 10 mm.

On the other hand, tracing fiber orientations in composites using ultrasound features has recently become a hot topic [19,20,21,22,23]. The existing inspection technologies can be categorized into two main directions, namely, the qualitive diagnosis of fiber waviness based on wave distortions in terms of time of flight and energy [24,25], and direct quantitative imaging of fiber orientations using instantaneous complex information of ultrasound (i.e., instantaneous amplitude, frequency and phase) [19,20,26,27,28]. Drinkwater [24] demonstrated that the use of the scattering information of ultrasound captured by an ultrasonic array has the potential to statistically evaluate the fiber waviness of different severities. Smith [19,26,29] proposed using the instantaneous amplitude, phase and frequency of ultrasound to characterize both in-plane and out-of-plane fiber waviness in a quantitative way for both unidirectional and woven composites. Recently, Yang [27,30] successfully developed a method for the assessment of 3D local fiber orientations in CFRP using planer ultrasound computed tomography and a Gabor-filter-based information diagram. Liu [31] proposed a double-side inverse-variance weight-synthetic method for the ultrasonic imaging of out-of-plane fiber wrinkling in thick CFRP and further expanded the detectable thickness range using ultrasound instantaneous information. Note that detailed material properties including material compliance matrix and ply thickness of the prepreg are required for the quantitative evaluation using ultrasound instantaneous information.

To sum up, the abovementioned UT technologies are mainly aimed at single-type defect inspection and cannot achieve the characterization and differentiation of mixed-type defects in thick CFRP. Fiber waviness (especially out-of-plane fiber waviness) and thick resin layers may coexist in thick composites [6]. Both defects can distort ultrasound propagation and result in variations in wave reflection and transmission. In addition, volumetric defects may interfere with instantaneous amplitude and phase features of ultrasound and cause fake images of fiber orientations constructed by instantaneous ultrasound information [3]. To provide accurate defect information for the processing parameter optimization of thick CFRP, identification between volumetric defects and fiber waviness in thick wavy composites using a multiple-frequency method [32] and ultrasound non-reciprocity [25,33] (manifested as time-of-flight difference when wave propagation direction is reversed) were proposed in our previous work. In this study, a novel imaging method of fiber waviness using probability-based ultrasound non-reciprocity with unknown material properties is proposed for defect characterization in thick composites.

In this manuscript, simulation and experiment setups for imaging diverse defects in thick composites using two phased array probes are first introduced. Subsequently, ultrasound propagation in non-wavy and wavy composites with 2 mm-diameter voids is investigated numerically. To gain an insight into the generation mechanism of ultrasound non-reciprocity, the time frequency spectra of the first-arrival transmitted waves in the thick composites with different microstructures are compared when the wave propagation directions are reserved. Subsequently, the time-of-flight difference (ToF Dif, i.e., ultrasound non-reciprocity) of transmitted ultrasound along different propagation paths between the two probes is calculated in both experiment and simulation. Probabilistic diagnostics using ToF Dif, reflected and transmitted energy of the ultrasound captured from the sensor network built by the two probes, are then adopted for the image diverse defects in the thick composites.

## 2. Simulation and Experiment Setup

**Figure 1:**
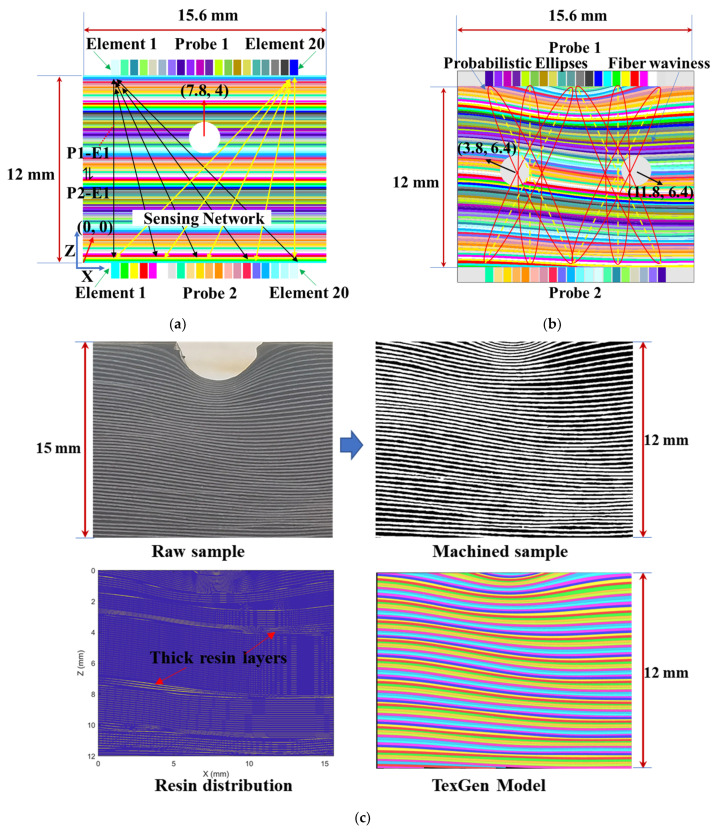

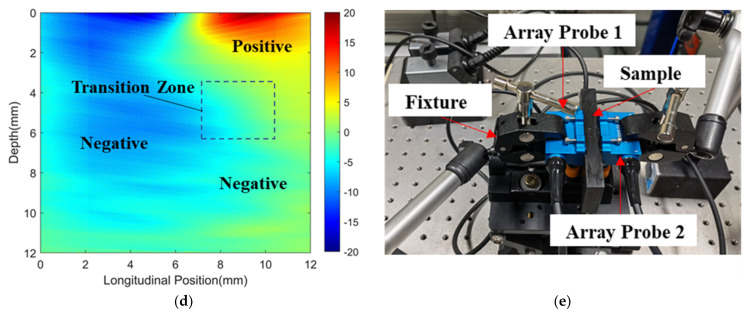
Numerical models of (**a**) non-wavy and (**b**) wavy composites with voids, (**c**) procedure of fiber orientation modeling, (**d**) fiber angle (indicated by color) distribution of (**c**), (**e**) experimental setup for data acquisition.

Two types of composite samples, namely, non-wavy (layup: [0/90]_24s_) and wavy composites (out-of-plane fiber waviness, layup: [0/90]_30s_), were fabricated using carbon-fiber-reinforced epoxy prepreg (T300-USN15000-7901-33%). The samples were cured for 2 h at 120 °C in a vacuum hot press machine. After curing, typical layer thickness for the non-wavy composites was measured as 0.125 mm. Half steel cylinders with diameters of 6 mm were placed on the layered prepreg during curing to generate out-of-plane fiber waviness [33], and the wavy composites were machined to the same thickness as that of the non-wavy composites (geometry: 200 mm (length) × 100 mm (width) × 12 mm (thickness)). Side-drilled holes (SDHs) of 2 mm diameters (refer to Figure 1a,b for the hole positions) were post-machined to represent volumetric defects in both the non-wavy and wavy samples.

Four types of numerical models, i.e., non-wavy and wavy composites with/without voids, were built, and the models of the porous non-wavy and wavy composites are shown in Figure 1a,b. To facilitate the understanding of wave interaction with fiber waviness, numerical models of the wavy composites were built in a cloud engineering simulation platform (OnScale^®^ 1.30.11.0) with accurately duplicated fiber orientations (as shown in Figure 1c) with an image processing method in MATLAB^®^ 2020a. The fiber angles relative to the horizontal direction within the 20-element range (element pitch 0.6 mm) of the numerical model in Figure 1c are shown in Figure 1d. Using a Python code script, the geometry of the wavy composites was constructed with the extracted fiber orientation information in an open source software (TexGen^®^ v3.12.0) developed by the University of Nottingham and exported to OnScale^®^ with the desired simulation settings.

A structural mesh with low-order quadrilateral elements was used in the 2D simulation. A Ricker wave with a center frequency of 6 MHz was adopted for the excitation. The excitation signal was generated by applying surface pressure at the center of the array elements, and the receiving signals were extracted as the average values of the surface pressure of a specific array element in the phased array probes. Considering the influence of thick resin layers (>10 μm, as shown in Figure 1c) on the wave transmission and trade-off between the simulation accuracy and efficiency, a mesh size of 10 μm (less than 1/30 wavelength of the longitudinal wave) was adopted. An absorption boundary was adopted on the left and right sides of the models to minimize boundary reflections. The composites were defined as linear elastic anisotropic material with mass density (1560 kg/m^3^) along with a compliance matrix (E_11_ = 161 GPa, E_22_ = E_33_ = 11.38 GPa, G_23_ = 3.96 GPa, G_12_ = G_13_ = 5.17 GPa, ν_23_ = 0.44, ν_12_ = ν_13_ = 0.32, where index 1 means fiber direction). The resin layers were defined as isotropic linear elastic material using density (1301 kg/m^3^), bulk modulus (4.67 GPa) and Poisson’s ratio (0.37). An explicit solver was used to calculate wave responses.

Two phased array probes (Doppler 5L64-0.6·10) with a nominal center frequency of 5 MHz were connected to a phased array system (PeakNDT LTPA 128/64PR) using a customized splitter, as shown in Figure 1e. Ultrasonic signals were excited at 6 MHz with a negative pulse to optimize the inspection of fiber waviness according to our previous study [33]. Full matrix capture (FMC) was conducted on the two probes to collect ultrasound signals propagating in the composites along different wave paths in a contact manner using a gel couplant. The sampling frequency of the phased array system was set as 100 MHz and the captured data were linearly interpolated to reach a resampling frequency of 1000 MHz. The excited voltages varied from 100 V to 200 V. 10 tests were repeated for each measurement, and the averaged waveforms were used for discussion in this study.

To avoid boundary reflections, the time of flight of the transmitted ultrasound was calculated using the propagation time of the first-arrival waves. Ultrasound non-reciprocity was quantified by $ToF\;Dif_{ij}$ when the wave propagation direction was reversed.

(1) $$ToF\;Dif_{ij}=|ToF_{ij}^{b}-ToF_{ji}^{t}|$$

$ToF_{ij}^{b}$ represents the ToF of the transmitted ultrasound excited at the bottom element i and received by the top element j, and $ToF_{ji}^{t}$ is the ToF when the wave propagation direction is reversed. Note that ToF in this study was calculated using the arrival time of the signal’s highest peak of the first wave packet in the time domain, so ultrasound propagating along oblique paths in the non-wavy composites did not show measurable non-reciprocity. As shown in Figure 1a,b, array elements (pitch distance is 0.6 mm) on the two probes form a sensing network and the whole sample area can be covered by using FMC technology. A revised probability-based diagnostic algorithm [34] using ultrasound non-reciprocity along different wave paths was developed to image fiber waviness as below:

(2) $$P(x,z)=\sum_{i=1}^{N}\sum_{j=1}^{N}ToF\;Dif_{ij}(x,z)\cdot W_{ij}$$

Here, P(x,z) is the presence probability of fiber waviness at a certain position (x,z), evaluated by ToF Dif. $ToF\;Dif_{ij}(x,z)$ is the time-of-flight difference along the wave path between element i on the bottom and element j on the top of the tested sample. The affected area of each wave path is an ellipse, as shown in Figure 1b, and the area can be described using Equation (3) as below:

(3) $$\frac{\sqrt{{\left(x-Ax\right)}^{2}+{\left(z-Az\right)}^{2}}+\sqrt{{\left(x-Rx\right)}^{2}+{\left(z-Rz\right)}^{2}}}{\sqrt{{\left(Ax-Rx\right)}^{2}+{\left(Az-Rz\right)}^{2}}}\leq \gamma$$

where (Ax,Az) is the position of the actuator element and (Rx,Rz) is the position of the receiver element. The original point of the model is shown in Figure 1a, and the pitch distance of the element is 0.6 mm. Therefore, the (Ax,Az) and (Rx,Rz) of all elements can be calculated. γ is the coefficient controlling the ellipse area, and γ=1.005 was obtained in this study after trial and error to achieve the optimal diagnosis resolution. To avoid the influence of system non-reciprocity on the imaging of fiber waviness, the threshold $ToF\;Dif_{ij}(x,z)$ of 0.002 μs was used when processing the experiment data.

(4) $$W_{ij}=\left\{\begin{array}{c} 1,\;\;\;\;\;\;\;ToF\;Dif_{ij}\geq 0.002\;\\ 0,\;\;\;\;\;\;\;ToF\;Dif_{ij}<0.002 \end{array}\right\}$$

## 3. Results and Discussions

**Figure 2:**
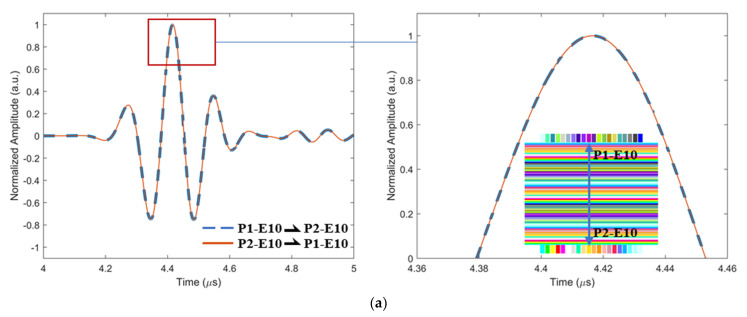

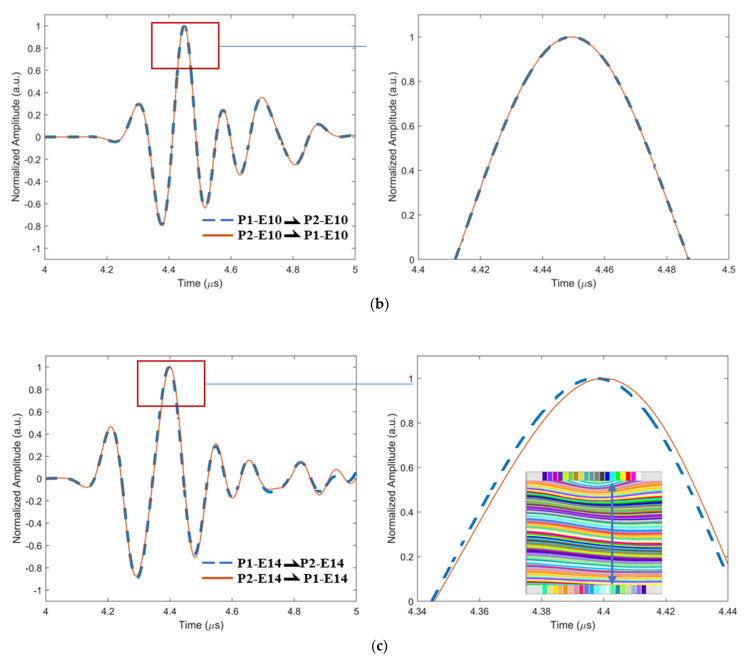
Simulated transmitted signals in the time domain along two propagation directions (**a**) between 10 elements in the intact composites, (**b**) between 10 elements in the porous composites with a void with a 2 mm diameter and (**c**) between 14 elements in the wavy composites.

**Figure 3:**
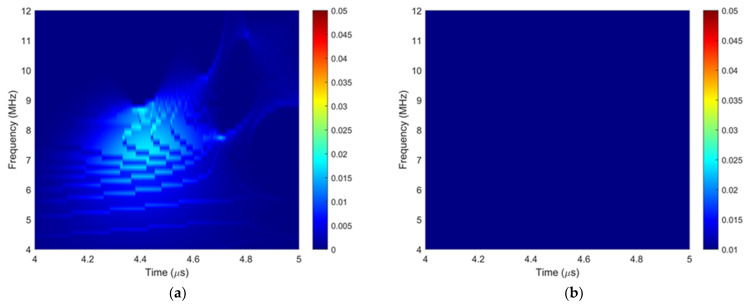

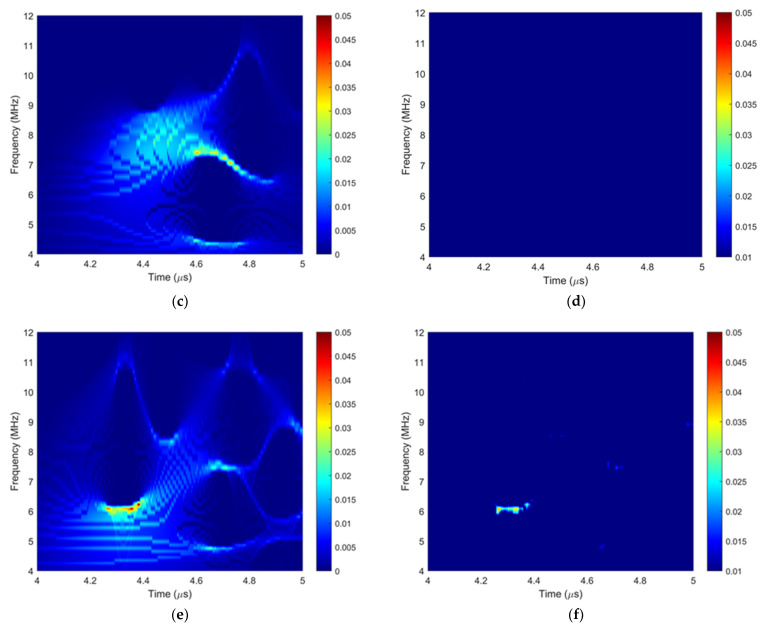
(**a**) Simulated time–frequency spectrum and (**b**) related difference in the transmitted signals between 10 elements in the intact composites, (**c**) simulated time–frequency spectrum, (**d**) related difference in the transmitted signals between 10 elements in the porous composites with a void with a 2 mm diameter and (**e**) simulated time–frequency spectrum and (**f**) related difference in the transmitted signals between 14 elements in the wavy composites.

To gain insight into the generation mechanisms of ultrasound non-reciprocity in composites, the ToF Dif of ultrasound propagating in the intact, porous and wavy composites was firstly investigated. Three pairs of transmitted signals along reversed directions captured from the three types of samples are shown in Figure 2. The legends P1-E10 and P2-E10 mean element 10 in the phased array probes 1 and 2. P1-E10 ⇀ P2-E10 means ultrasound excited on P1-E10 and received on P2-E10. Other legends in Figure 2 were named using the same principle. From comparison, fiber waviness generates noticeable ToF Dif between the two transmitted signals, as shown in Figure 2c, while no measurable ToF Dif is present in the other two cases, as shown in Figure 2a,b. To further explore the cause of ultrasound non-reciprocity in the wavy composites, the time–frequency spectra of the transmitted ultrasound in the three types of composites were comparatively calculated using squeezed wavelet transform [35], and they are displayed in Figure 3a,c,e (color bar indicates signal magnitude). Taking the time–frequency spectra of the transmitted signal in the intact sample as a reference (Figure 3a), fiber waviness (Figure 3c) introduces larger changes in the time–frequency features of the transmitted signals compared to voids (Figure 3b). Compared to Figure 3a, high-frequency components in the 9–11 MHz range are produced, while low-frequency components in the 6–9 MHz range reduce for the first-arrival wave (4.1–4.5 μs), as shown in Figure 3c. Such changes in the frequency distributions can be attributed to frequency-dependent transmission coefficients of the ultrasound at different frequencies in the anisotropic layered structure. Variations in the fiber orientations and the presence of thick resin layers change the local material properties (e.g., density, stiffness and layer thickness); consequently, the transmission coefficients of the ultrasound at different frequencies in the wavy composites differ from those of non-wavy composites. No observable non-reciprocity in the transmitted ultrasound was present for either the intact composites (Figure 3b) or the porous composites (Figure 3d). While fiber waviness induces measurable non-reciprocity in the time–frequency spectrum of the transmitted signal (Figure 3f), fiber waviness induces unsymmetrical changes in the material properties; therefore, the frequency-dependent transmission coefficients of ultrasound become different when the propagation direction is reversed. Due to the dispersion behaviors of ultrasound in anisotropic composites, ultrasound non-reciprocity manifesting as a difference in group velocity is generated in the wavy composites.

**Figure 4:**
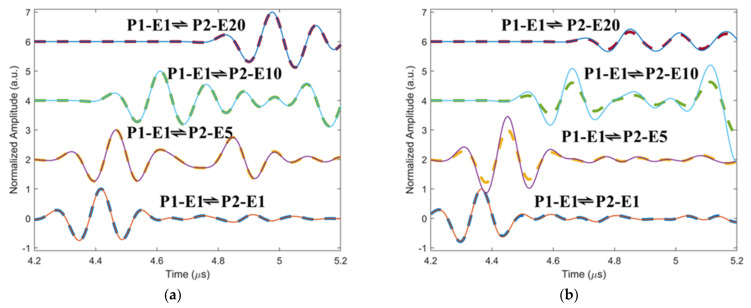
Simulated time–domain signals of ultrasound propagating in the (**a**) intact and (**b**) wavy composites along different wave paths; the solid lines represent signals propagating downwards, and the dashed lines are for signals propagating upwards.

To validate the effectiveness of the senor network built by the two phased array probes for the inspection of fiber waviness, simulated ultrasound signals propagating along different directions in the non-wavy and wavy composites without voids are shown in Figure 4a and Figure 4b, respectively. The legends P1-E1 and P2-E1 mean element 1 in the phased array probes 1 and 2, as shown in Figure 1a,b. P1-E1 ⇌ P2-E1 means signals transmitting between the element 1 of the two probes. The solid lines represent signals propagating downwards, and the dashed lines are for signals propagating upwards. The waveforms of the two signals propagating in different directions along the same wave path within the non-wavy composites remain the same regardless of the propagation angles relative to the fibers. Therefore, inclined interaction angles between ultrasound and straight fibers are not the cause of ultrasound non-reciprocity. However, for ultrasound propagating in the wavy composites, a noticeable difference can be observed in the waveforms when the wave propagation direction is reversed. Therefore, the fiber angle gradient induced by fiber waviness is responsible for the generation of ultrasound non-reciprocity, as shown in Figure 1d.

From Figure 2, Figure 3 and Figure 4, ultrasound non-reciprocity was proven to be sensitive to fiber waviness in the thick composites. In the following sections, ultrasound features including reflected and transmitted wave energy and ultrasound non-reciprocity are comparatively adopted to image diverse defects in the thick composites.

### 3.1. Defect Imaging and Differentiation in Non-Wavy Composites

**Figure 5:**
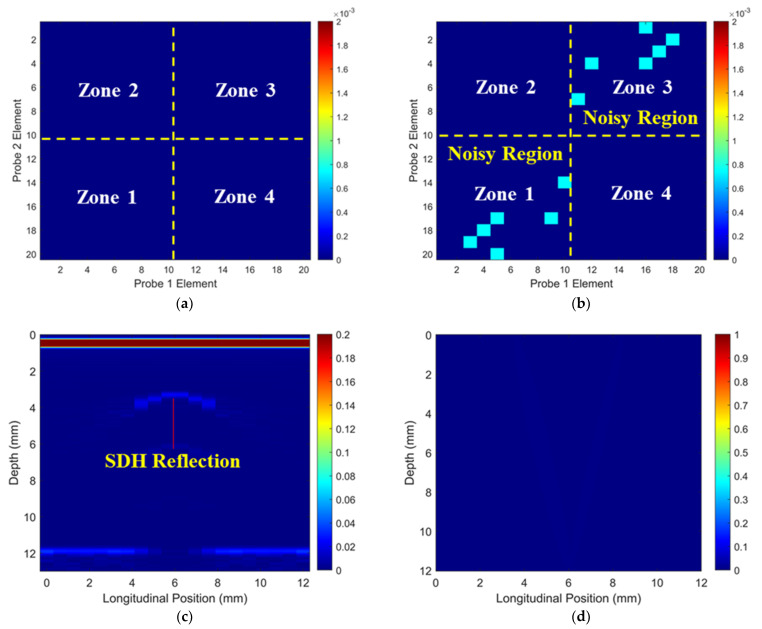
Simulated ToF Dif of ultrasound propagating in the non-wavy composites (**a**) without and (**b**) with a void with a 2 mm diameter, ultrasonic B-scans constructed by (**c**) reflected energy and (**d**) ToF Dif of the porous composites.

The simulated ToF Dif of the ultrasound propagating between different elements in the two probes within the non-wavy composites without and with a 2 mm-diameter void were calculated, and they are shown in Figure 5a and Figure 5b, respectively. Large voids induce slight ultrasound non-reciprocity when ultrasound propagates at large angles relative to the fibers, as shown in Zones 1 and 3 in Figure 5b. For ultrasound propagating within a 10-element range (i.e., Zones 2 and 4), no observable ToF Dif presents in either case. Ultrasonic B-scans constructed by reflected ultrasound energy and ToF Dif for the non-wavy composites with a 2 mm-diameter void are shown in Figure 5c,d. Two sets of FMC data acquired from 10 elements of the two probes (i.e., elements 1–11 and elements 10–20) were adopted to generate Figure 5d using the probability-based diagnostic algorithm (Equation (2)). The void (created by a 2 mm-diameter SDH) only generates clear indications in the traditional B-scan, as shown in Figure 5c.

**Figure 6:**
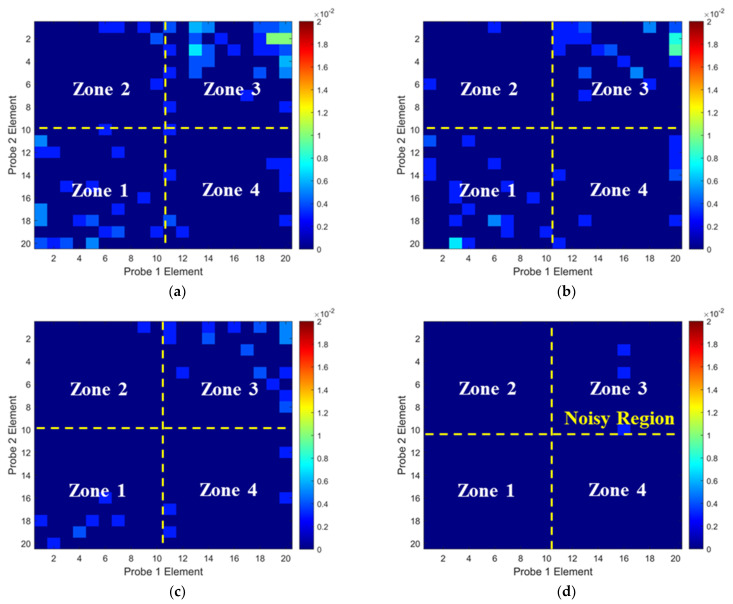
Experimental ToF Dif of transmitted ultrasound captured from the intact composites under excitation voltages of: (**a**) 100 V, (**b**) 140 V, (**c**) 180 V and (**d**) 200 V.

**Figure 7:**
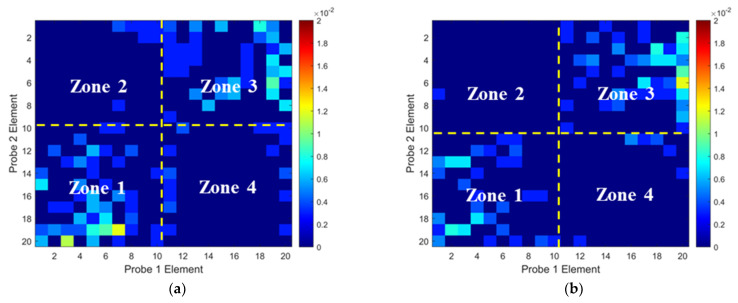

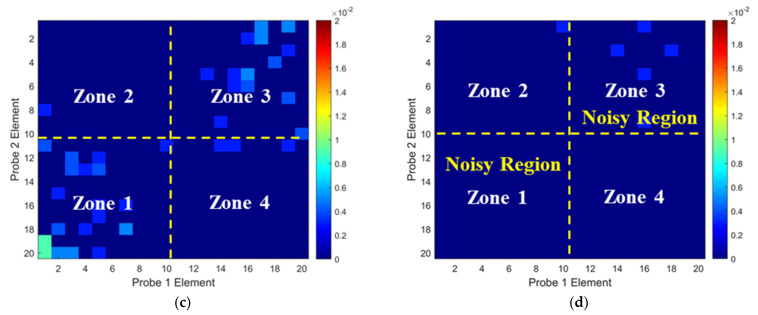
Experimental ToF Dif of transmitted ultrasound captured from the porous composites with a void of 2 mm diameter under excitation voltages of: (**a**) 100 V, (**b**) 140 V, (**c**) 180 V and (**d**) 200 V.

**Figure 8:**
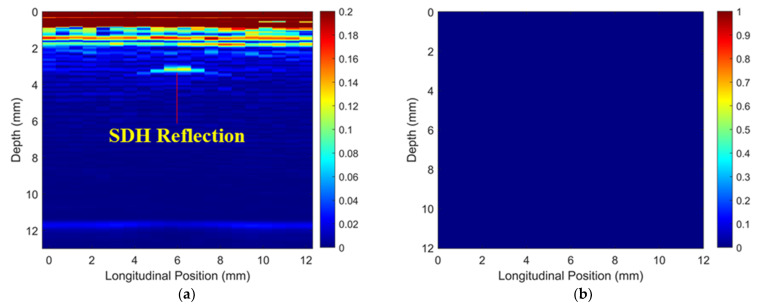
Experimental ultrasonic B-scans constructed by (**a**) reflected energy and (**b**) ToF Dif of the porous composites with a void of 2 mm diameter.

To validate the numerical predictions, the non-wavy composites without and with a 2 mm-diameter SDH were inspected experimentally. Firstly, the influence of excitation voltages on the ultrasound non-reciprocity was investigated. The experimental ToF Dif of ultrasound propagating within the non-wavy composites without and with a 2 mm-diameter void under different excitation voltages was calculated and is shown in Figure 6 and Figure 7, respectively. Under low-excitation voltages below 180 V, the measurable ToF Dif present in Zone 2 and 4 for both samples and the ultrasound non-reciprocity caused by the measurement system disappear when the voltages reach 200 V. This is because the signal-to-noise ratio of the transmitted ultrasound excited by low voltages is relatively low, which leads to calculation errors in the ToF of the first-arrival wave. Therefore, the excitation voltage of 200 V is recommended to minimize the ultrasound non-reciprocity induced by the measurement system. Experimental ultrasonic B-scans constructed by reflected ultrasound energy and ToF Dif for the non-wavy composites with a 2 mm-diameter void are shown in Figure 8a,b. Experimental results shown in Figure 8 match well with the numerical predications shown in Figure 5. To sum up, by calculating ToF in the time domain and optimizing the number of elements adopted for FMC data processing, large voids with a 2 mm diameter did not generate indications in the B-scans constructed by ToF Dif using the proposed probability-based diagnostic algorithm.

### 3.2. Defect Imaging and Differentiation in Wavy Composites

**Figure 9:**
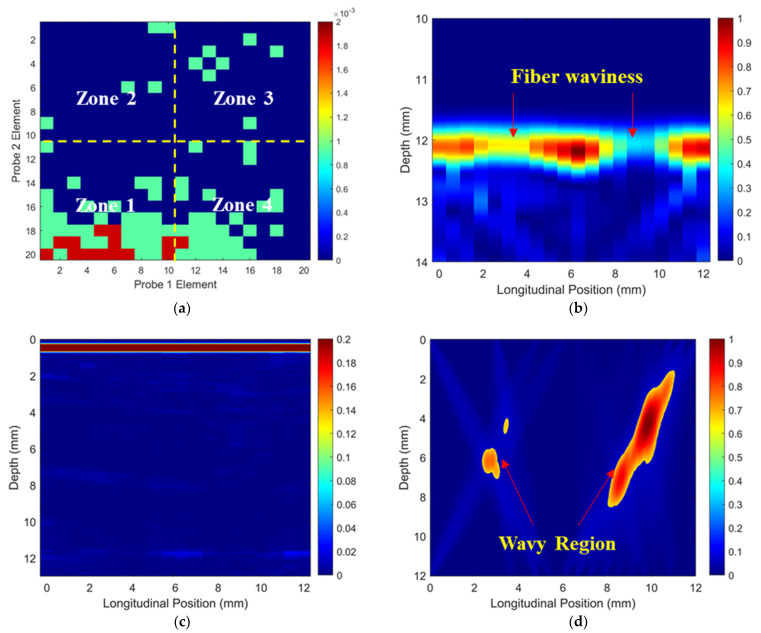
(**a**) Simulated ToF Dif and (**b**) transmitted ultrasound energy, ultrasonic B-scans constructed by (**c**) reflected energy and (**d**) ToF Dif of the wavy composites without voids.

Simulated ToF Dif of ultrasound propagating in the wavy composites without voids were calculated and are shown in Figure 9a. Distinct from Figure 5a,b, a non-zero ToF Dif is present in both Zones 2 and 4, i.e., fiber waviness induces measurable ultrasound non-reciprocity. The ultrasound-transmitted energy between different elements of the two probes along the vertical direction is shown in Figure 9b. The energy loss caused by fiber waviness in two main regions is present (i.e., 2–4 mm and 8–10 mm in the longitudinal direction) due to the scattering and deflection of the ultrasound after passing through the wavy region. Ultrasonic B-scans constructed by reflected ultrasound energy and ToF Dif are displayed in Figure 9c and Figure 9d, respectively. Fiber waviness does not produce clear indications in the traditional B-scan image in Figure 9c, while the proposed probability-based diagnostic algorithm successfully images the wavy region. The position and intensity of the energy loss of the transmitted signals in Figure 9b match well with those of the wavy region in Figure 9d. In addition, the detected wavy region in Figure 9d (depth range: 4–6 mm and longitudinal position: 8–10 mm) agrees with the transition zone of the fiber angles in Figure 1d. Therefore, the proposed method is sensitive to the transition region of fiber waviness, where there is a drastic change in fiber angle from positive to negative values.

**Figure 10:**
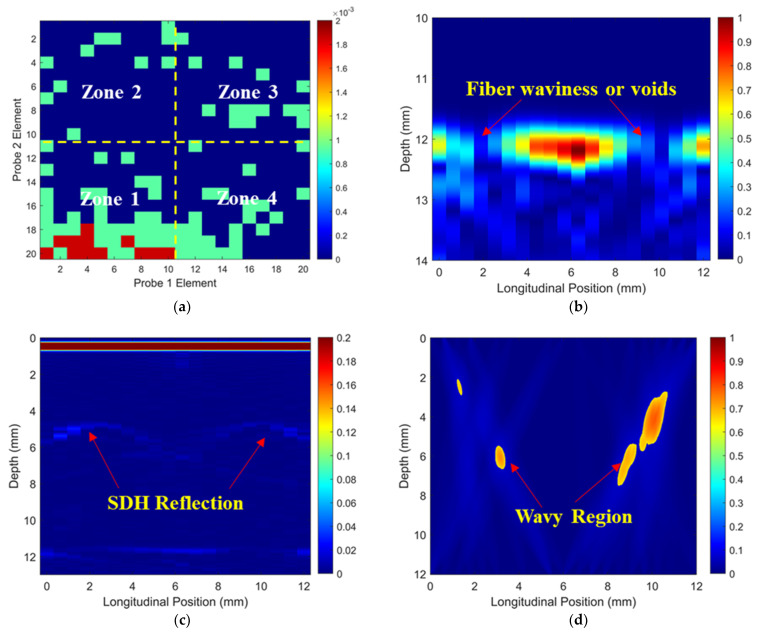
(**a**) Simulated ToF Dif and (**b**) transmitted ultrasound energy, ultrasonic B-scans constructed by (**c**) reflected energy and (**d**) ToF Dif of the wavy composites with two voids with a 2 mm diameter.

To further investigate the effectiveness of the proposed method for imaging fiber waviness in thick wavy composites with large voids, as shown in Figure 1b, the simulated ToF Dif of the ultrasound along different wave paths is shown in Figure 10a. Similar to Figure 9a, a non-zero ToF Dif is present in Zones 2 and 4 in Figure 10a. The two voids further cause a greater energy loss of transmitted ultrasound in Figure 10b compared to that in Figure 9b. Therefore, fiber waviness and voids cannot be differentiated based on the reductions in transmitted energy. Reflections from the two 2 mm-diameter voids can be observed in the traditional B-scan in Figure 9c. Due to wave deflection and scattering caused by fiber waviness, the profiles of the two voids are distorted. Fiber waviness in the wavy porous composites is successfully imaged in Figure 10d. Due to the removal of wavy region caused by the voids, the area of the wavy region in Figure 10d is reduced compared to that in Figure 9d. In addition, fiber waviness mainly presents on the right side, which also matches well with the results shown in Figure 9d.

**Figure 11:**
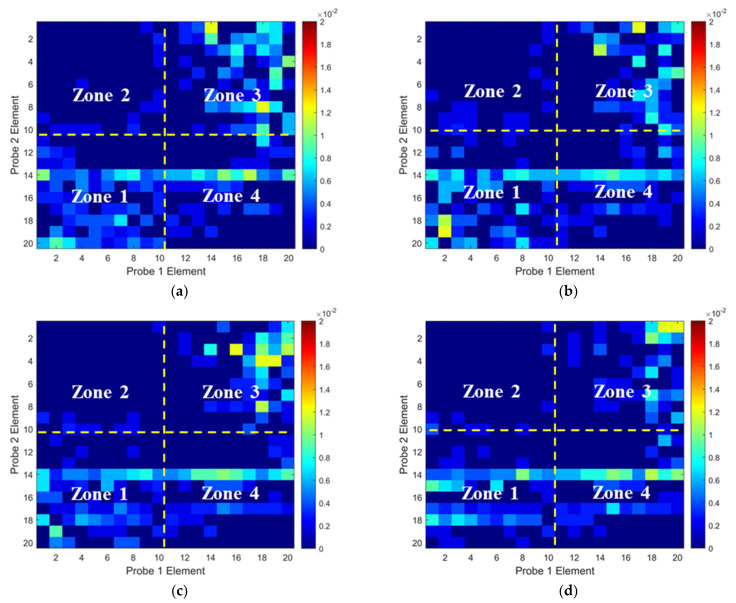
Experimental ToF Dif of transmitted ultrasound captured from wavy composites without voids under excitation voltages of (**a**) 100 V, (**b**) 140 V, (**c**) 180 V and (**d**) 200 V.

**Figure 12:**
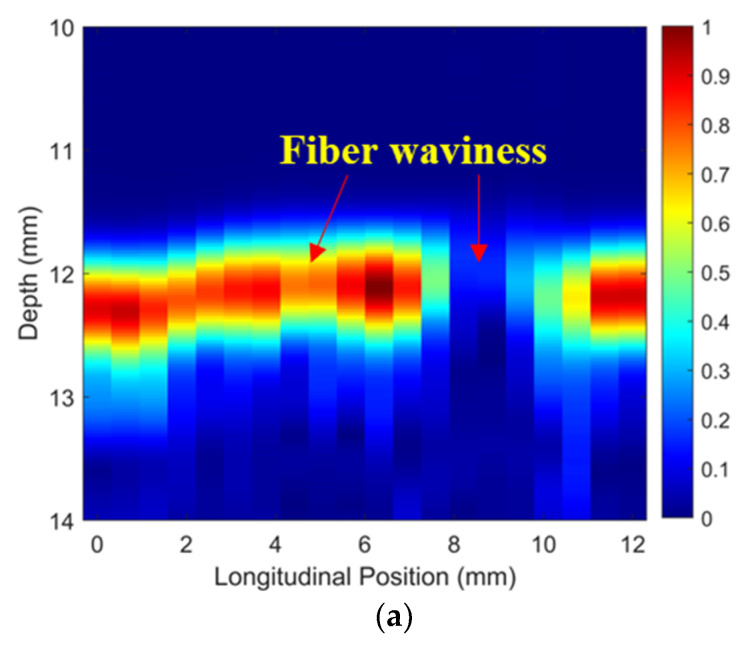

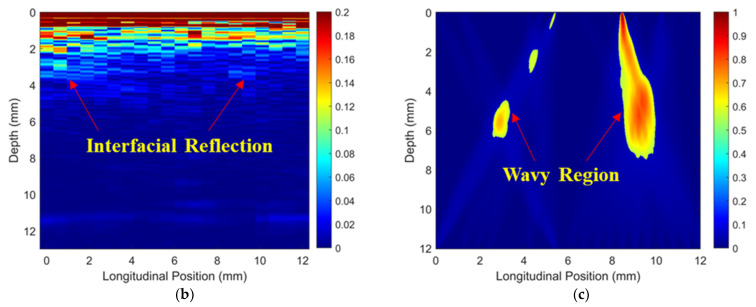
(**a**) Experimental transmitted ultrasound energy, ultrasonic B-scans constructed by (**b**) reflected energy and (**c**) ToF Dif of the wavy composites without voids.

To validate the numerical predictions as shown in Figure 9 and Figure 10, an inspection of the thick wavy composites using the two phased array probes was conducted. The experimental ToF Dif of the ultrasound propagating in the wavy composites without voids under different excitation voltages is shown in Figure 11. A non-zero ToF Dif is observed in Zones 2 and 4 in all four scenarios. In Figure 11d, fiber waviness induces significant ultrasound non-reciprocity, especially in Zone 4, compared to voids, as shown in Figure 7d. Similar to the numerical predictions in Figure 9b, a significant energy loss is observed between 8 and 10 mm in the longitudinal direction in Figure 12a, which indicates the presence of severe defects. However, in the conventional B-scan in Figure 12b, there is no obvious evidence of fiber waviness, but reflections from the thick resin layers are present, while fiber waviness is successfully imaged using ultrasound non-reciprocity, as shown in Figure 12c. From the comparison between Figure 12a and Figure 12c, the defect positions of the fiber waviness match well in the longitudinal direction and also agree with the simulation results, as shown in Figure 9. Therefore, the proposed probability-based method using non-reciprocity can provide the distribution of fiber waviness in the depth and longitudinal directions.

**Figure 13:**
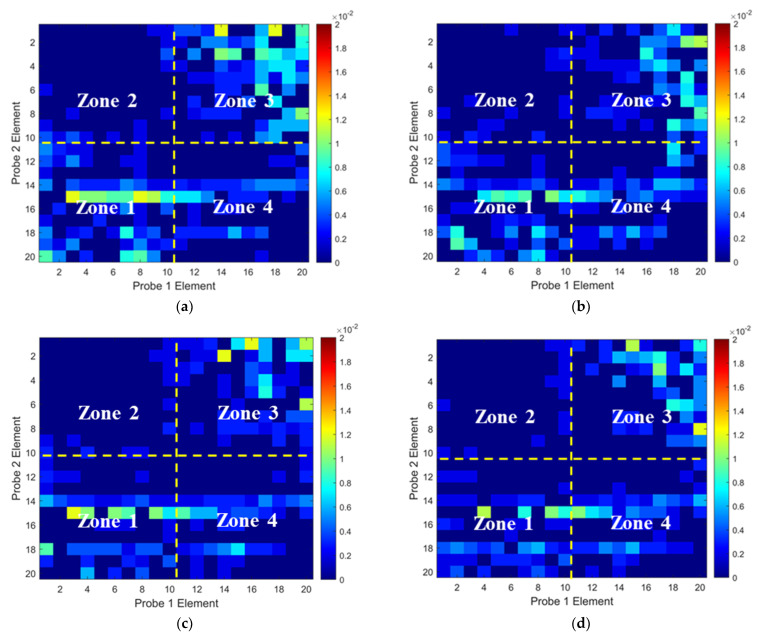
Experimental ToF Dif of transmitted ultrasound captured from wavy composites with two voids with a 2 mm diameter under excitation voltages of (**a**) 100 V, (**b**) 140 V, (**c**) 180 V and (**d**) 200 V.

**Figure 14:**
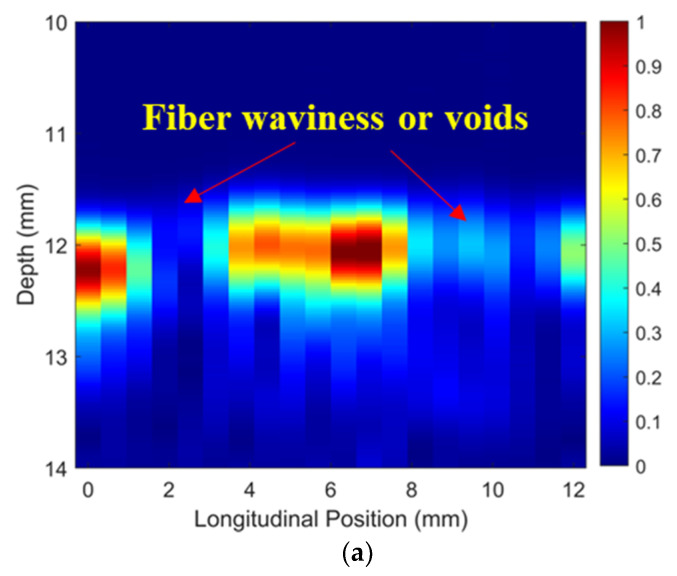

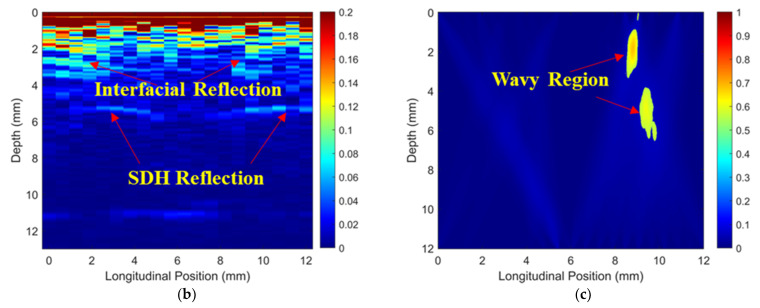
(**a**) Experimental transmitted ultrasound energy, ultrasonic B-scans constructed by (**b**) reflected energy and (**c**) ToF Dif of the wavy composites with two voids with 2 mm diameters.

The experimental ToF Dif of the ultrasound propagating in the wavy composites with a void with a 2 mm diameter under different excitation voltages is shown in Figure 13. A non-zero ToF Dif is also present in Zones 2 and 4 in Figure 13a–d. The maximum value of ToF Dif is reduced under 200 V excitation, while a non-zero ToF Dif is produced at more positions in Zone 4 in Figure 13d, compared to the wavy composites without voids, as shown in Figure 11d. This is caused by changes in the microstructures of the composites due to the removal of the wavy region by the voids. Because of wave scattering induced by the voids, more energy loss of transmitted signals in the longitudinal position is present in Figure 14a compared to that in Figure 12a. In the traditional ultrasonic B-scan in Figure 14b, indications of the two voids can be observed with some distorted profiles. However, such reflections cannot be differentiated from near-surface interfacial reflections from the thick resin layers, as highlighted in Figure 14b, without prior knowledge of the defect location. Similar to the simulation results, as shown in Figure 10d, the area of the indications of fiber waviness in Figure 14c is reduced compared to that for the wavy composites without voids in Figure 12c. Through both simulation and experiment, the proposed method is demonstrated to be capable of imaging fiber waviness in thick composites regardless of voids. With the combination of changes in the transmitted and reflected wave energy, and ultrasound non-reciprocity, diverse defects including volumetric defects and fiber waviness can be characterized with detailed defect type, shape and position.

## 4. Conclusions

In this manuscript, a novel method for imaging fiber waviness in thick composites using ultrasound non-reciprocity with a probability-based diagnostic algorithm was proposed. Conclusions can be drawn from the simulation and experiment as below:

- Ultrasonic images of large voids with a 2 mm diameter in traditional B-scans may be distorted by fiber waviness and masked by reflections from the thick resin layers near the surface. The presence of fiber waviness cannot be determined in thick composites with diverse defects using traditional pulse echo or through transmission testing.
- Fiber waviness introduces difference in the transmission coefficients of ultrasound under distinct frequencies when the propagation direction is reversed. Due to dispersion behaviors of ultrasound in composites, ultrasound non-reciprocity in terms of group velocity generates in the wavy composites. Such ultrasound non-reciprocity is sensitive to fiber angle gradient caused by fiber waviness.
- The proposed probability-based diagnostic algorithm on ultrasound non-reciprocity successfully imaged and identified fiber waviness in thick wavy composites regardless of the presence of voids. With the combination of transmitted and reflected wave energy, the proposed method can improve the reliability of the ultrasonic characterization of diverse defects in the thick composites.

As a proof-of-concept study, the proposed method was proven to be capable of imaging fiber waviness in thick wavy composites. However, theoretical models are required to establish a quantitative relationship between the ultrasound non-reciprocity and severity of fiber waviness. Therefore, the coefficient γ for the quantitative evaluation of fiber waviness using the probability-based ultrasound non-reciprocity can be optimized. Furthermore, future work will be devoted to signal processing to improve the sensitivity of the proposed method for fiber waviness at small deviation angles.

## Data Availability

Not applicable.
